# Yeast mannoproteins are expected to be a novel potential functional food for attenuation of obesity and modulation of gut microbiota

**DOI:** 10.3389/fnut.2022.1019344

**Published:** 2022-10-14

**Authors:** Xiang Li, Junsong Wu, Yijun Kang, Dan Chen, Guijie Chen, Xiaoxiong Zeng, Jialian Wang

**Affiliations:** ^1^School of Marine and Biological Engineering, Yancheng Teachers’ University, Yancheng, China; ^2^Department of Basic Medical Science, Jiangsu Vocational College of Medicine, Yancheng, China; ^3^College of Food Science and Technology, Nanjing Agricultural University, Nanjing, China

**Keywords:** yeast, mannoproteins, obesity, gut microbiota, *Parabacteroides distasonis*, *Lactobacillus*

## Abstract

The yeast mannoproteins (MPs), a major component of yeast cell walls with large exploration potentiality, have been attracting increasing attention due to their beneficial effects. However, the information about the anti-obesogenic activity of MPs is still limited. Thus, the effects of MPs on the high-fat diet (HFD)-induced obesity and dysbiosis of gut microbiota were investigated in this work. The results showed that MPs could significantly attenuate the HFD-induced higher body weight, fat accumulation, liver steatosis, and damage. Simultaneously, the inflammation in HFD-induced mice was also ameliorated by MPs. The pyrosequencing analysis showed that intervention by MPs could lead to an obvious change in the structure of gut microbiota. Furthermore, the prevention of obesity by MPs is highly linked to the promotion of *Parabacteroides distasonis* (increased from 0.39 ± 0.12% to 2.10 ± 0.20%) and inhibition of *Lactobacillus* (decreased from 19.99 ± 3.94% to 2.68 ± 0.77%). Moreover, the increased level of acetate (increased from 3.28 ± 0.22 mmol/g to 7.84 ± 0.96 mmol/g) and activation of G protein-coupled receptors (GPRs) by MPs may also contribute to the prevention of obesity. Thus, our preliminary findings revealed that MPs from yeast could be explored as potential prebiotics to modulate the gut microbiota and prevent HFD-induced obesity.

## Introduction

Obesity, recognized as a disease with serious morbidity and increased mortality, has dramatically spread throughout the developed and developing countries due to a shift to diets, including addictive and/or high-calorie foods and lack of exercise (1, 2). In the past four decades, the prevalence of obesity and overweight has nearly tripled worldwide, which has become a growing public health challenge of the twenty-first century (3). Furthermore, obesity may lead to an increased risk of other obesity-related metabolic disorders, such as non-alcoholic fatty liver disease (NAFLD), type 2 diabetes (T2DM), and cardiovascular diseases (CDs) (4, 5). Obesity is a chronic and progressive process with multi-factorial factors and complex interactions, including physiological, sociopolitical, behavioral, and environmental factors (3, 6). Although the molecular mechanism for obesity is still not fully understood, obesity essentially represents a long-term positive imbalance between energy intake and energy expenditure, thereby increasing body fat (7). Pharmaceutical drugs, such as orlistat, could prevent, and treat obesity, however, its adverse effects, including acute kidney injury, subacute liver failure, and gastrointestinal adverse effects, block its further application (8). Thus, a potential novel therapeutic strategy for the prevention and treatment of obesity is still highly needed.

In recent decades, the effect of the gut microbiota on obesity has attracted much attention due to its key role in calorie harvest, energy homeostasis, and regulation of fat storage (9, 10). Recently and more strikingly, the experiments using germ-free mice and fecal microbiota transplantation have demonstrated the causality between the gut microbiota and the development of obesity (11, 12). More specifically, some key beneficial gut microbiota responsible for the prevention of obesity, such as *Akkermansia muciniphila* (13), *Parabacteroides distasonis* (14), and *Dysosmobacter welbionis* (15), and pathogenic bacteria which could promote obesity, such as *Erysipelatoclostridium Ramosum* (16) and *Enterobacter cloacae* B29 (17), have been identified, separated, and verified at the species level. The gut microbiota is expected to be a novel therapeutic target for the prevention and treatment of obesity. Thus, a series of microbiota-targeted diets are presented and evaluated with the growing public awareness of the gut microbiota (18, 19). Thereinto, dietary polysaccharides, which served as potential prebiotics, have recently emerged with the growing public awareness of their probiotic effect on gut microbiota (20).

Yeast is an important food resource used for fermentation in the food industry, and a large amount of yeast by-products is available every year (21). The yeast cell wall is mainly composed of β-glucan (60%) and mannoproteins (MPs, 40%), making them a potential source for providing functional ingredients (22). Nowadays, yeast by-products are mainly processed into animal feed or used to produce β-glucan (22, 23). β-Glucan from yeast has been widely investigated, whereas MPs from yeast attract much less attention. MPs are highly glycosylated proteins with molecular weights ranging from 20 to 200 kDa, containing 80–90% of carbohydrates and 5–20% of protein, and the potential structure of MPs has been reported in previous work (22, 24, 25). In recent years, the MPs have attracted more and more attention due to their alleged health-promoting functions, such as stimulation of angiogenesis, immunoactivities, and antineoplastic activities (25–27). However, the effects of MPs on obesity and gut microbiota are still unknown. Thus, the aim of the present work was to evaluate the potential anti-obesogenic effect of MPs on a high-fat diet (HFD)-induced obesity. Furthermore, the role of gut microbiota in the prevention of obesity by MPs was also investigated.

## Materials and methods

### Materials

The MPs from *Saccharomyces cerevisiae* were kindly provided by Angel Nutritech Co., Ltd. (China). The MPs were prepared according to the previous work with some modifications (25, 26). Briefly, after being sieved and purified, the slurry of *S. cerevisiae* cells was mixed with 3% sodium chloride, and the solution was incubated at 55°C for 24 h with agitation at 120 rpm/min. The residual autolyzed cells were obtained by centrifugation at 5,000 g for 10 min, and the MPs were extracted by water at 121°C for 4 h. The supernatant was collected and mixed with a triple volume of absolute alcohol. After keeping at 4°C for one night, the precipitated MPs were collected and deproteinized using the trichloroacetic acid method. The obtained solution was further mixed with a triple volume of absolute alcohol. The precipitate was collected, dissolved in distilled water, and further separated by Sepharose CL-4B to obtain the purified MPs. The contents of carbohydrates and protein were 86.3 ± 2.37% and 14.6 ± 1.45%, respectively. The molecular weight of MPs was 78 kD. The mice diets, including D12450J with 10 kcal% fat and D12492 with 60 kcal% fat, were purchased from Research Diets, Inc. (New Brunswick, NJ, USA).

### Mice and diets

Six-week-old C57BL/6 male mice (*n* = 24, Shanghai SLAC Laboratory Animal Co., Ltd., Shanghai, China) were bred in the Animal Center of Nanjing Agricultural University (SYXK < Jiangsu > 2011-0037). All animal experimental protocols in this work were approved by the Institutional Animal Ethics Committee of the Experimental Animal Center of Nanjing Agricultural University. The mice were housed in specific pathogen-free (SPF) animal rooms under a 12-h dark-light cycle with *ad libitum* access to food and water. After an accommodation period of 1 week, mice were randomly divided into three groups (*n* = 8 per group), and fed for 10 weeks with a normal-chow diet (D12450J, coded as ND group), HFD (D12492, coded as HFD group), and HFD plus daily yeast MPs with a dosage of 400 mg/kg of body weight (coded as HFD-MP). The dosage of MPs in this work was chosen according to the previous work (23). Mice were supplemented daily with 0.2 mL of water in the ND and HFD groups and 0.2 mL of MPs solution (400 mg of MP was dissolved in 10 mL of sterilized water) in the HFD-MP group by intragastric gavage. The body weight and food intake were assessed on a weekly basis. After overnight fasting, mice were anesthetized using carbon dioxide and then euthanized by cardiac puncture at the end of 10 weeks. The blood was drawn in tubes containing EDTA and centrifuged at 4,000 g to obtain plasma. The adipose tissue and liver were obtained and weighed. A part of the epididymis fat and liver samples were fixed with a 4% of paraformaldehyde solution. After embedding in paraffin, the epididymis fat and liver samples were sectioned and stained with hematoxylin and eosin (H&E stain). Then, the slices were observed under a light microscope.

### Biochemical analysis

The plasma levels of triglycerides (TGs), low-density lipoprotein cholesterol (LDL-C), total cholesterol (TC), fasting plasma glucose, and alanine transaminase (ALT) were detected by a commercial kit according to the manufacturer’s instructions. The plasma interleukin-1β (IL-1β), IL-10, IL-6, and tumor necrosis factor-alpha (TNF-α) levels were evaluated by commercial ELISA kits from Neobioscience Biological Technology Co., Ltd. (Shenzhen, China).

### Gut microbiota analysis by 16S rRNA gene sequencing

The genomic DNA was extracted from the feces of the mice using the QiAamp DNA Stool Mini Kit (no. 51504, Qiagen, Germany). The V3-V4 region was amplified from purified DNA with the primers 341F (CCTACGGGNGGCWGCAG) and 805R (GACTACHVGGGTATCTAATCC). Sequencing was performed at an Illumina MiSeq platform by DeepBiome Co., Ltd. (Jinan, China) based on the manufacturer’s guidelines to obtain the raw fastq files. The quality filtering of data was carried out using Trimmomatic (version 0.36). The paired reads were merged by USEARCH (version 11.2.64)^1^ using the default parameters. The zero-radius operational taxonomic unit (ZOTU) was obtained using USEARCH. The bioinformatic analysis was performed by a previously reported method (28).

### Short-chain fatty acid analyses

The levels of short-chain fatty acids (SCFAs) in mice cecal contents, including acetic, propionic, and n-butyric, were analyzed by gas chromatography (GC, 6890 N, Agilent) equipped with flame ionization detector (FID) and HP-INNOWAX capillary column (30 m × 0.25 mm × 0.25 μm, Agilent) using 2-ethylbutyric acid as internal standard (29). Briefly, the distilled water was added to cecal contents at a ratio of 1:5 (w/v). After centrifugation, the samples were mixed with internal standard solution (0.3 mg/mL of 2-ethylbutyric acid prepared in 0.2 mol/L of HCl solution) in equal volumes to obtain the solutions for GC analysis. The conditions of GC analysis were described in the previous work (29).

### Ribonucleic acid extraction and quantification of gene expression

The total ribonucleic acid (RNA) in liver tissue was extracted by TaKaRa MiniBEST Universal RNA Extraction Kit (TaKaRa Bio. Inc., Beijing, China). The RNA was diluted and reverse-transcribed to cDNA by PrimeScript RT Master Mix (TaKaRa) after quantifying by using NanoDrop 2000 Spectrophotometer (Thermo Fisher Scientific Inc., Waltham, MA, USA). Then, the cDNA was used for RT-qPCR analysis using SYBR Green Master Mix on a QuantStudio 6 Flex (Thermo Fisher Scientific Inc.). The glyceraldehyde-3-phosphate dehydrogenase (GAPDH) was used as a housekeeping gene, and the mRNA expression was calculated using the 2^–ΔΔCt^ method. The specific primers used are summarized in Supplementary Table 1.

### Statistical analysis

The outliers in this work were checked by GraphPad Prism 9.3.1 based on the Grubbs test (San Diego, CA, USA). The data were presented as mean ± SEM. The normality of all data was checked by SPSS 22 software (IBM) according to Shapiro–Wilk test. If the data for multiple-group comparisons had normal distribution, the statistical significance was performed by SPSS 22 software (IBM) using the one-way ANOVA procedure followed by the Tukey test; otherwise, the statistical significance was calculated using the Mann–Whitney test. The relationship between the parameters of obesity and gut microbiota was analyzed by Spearman’s correlation analysis using SPSS 22 software using the data of all samples in ND, HFD, and HFD-MP groups (24 samples). All results were considered statistically significant at *p* < 0.05.

## Results

### Mannoproteins treatment ameliorated obesity and liver steatosis in high-fat diet-induced obese mice

**FIGURE 1:**
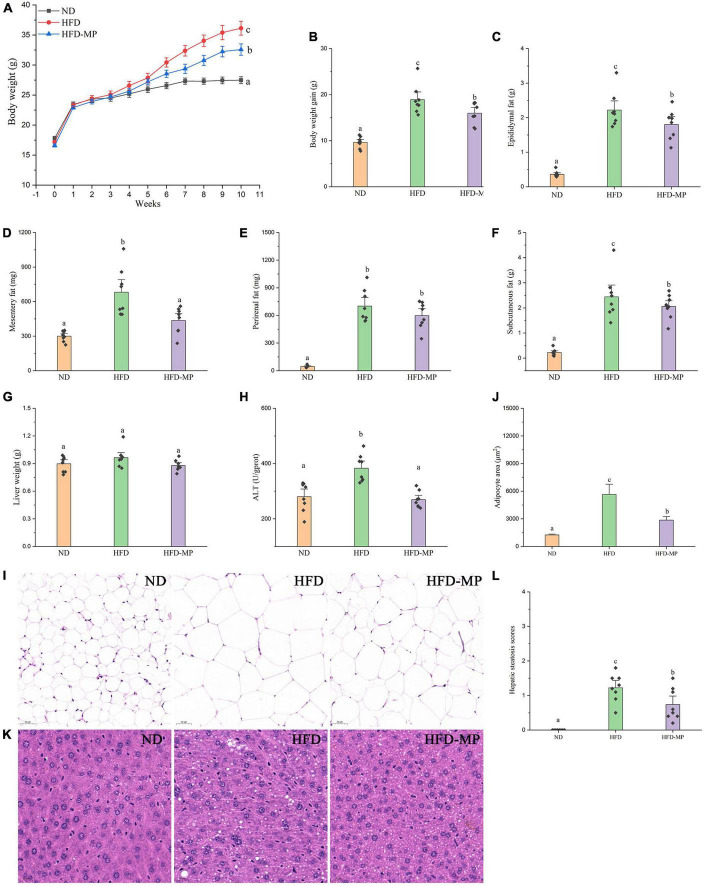
Impact of HFD and MP intervention on body features of mice. **(A)** Body weight, **(B)** body weight gain, weights of **(C)** epididymal, **(D)** mesentery, **(E)** perirenal, **(F)** subcutaneous fat pads, **(G)** liver weight, **(H)** plasma alanine transaminase (ALT) level, epididymal adipocyte sections after H&E staining **(I)**, quantification of adipocyte area by ImageJ software **(J)**, liver **(K)** sections after H&E staining, and hepatic steatosis scores **(L)**. The data are represented as the mean ± SEM. Statistical differences were carried out by one-way ANOVA followed by Turkey’s test, *p* < 0.05 indicates significant differences.

As shown in Figure 1, HFD significantly resulted in the obesity of mice by increasing the body weight, promoting the accumulation of white adipose tissue, and inducing hepatic lipid accumulation and steatosis. MP treatment could significantly decrease body weight gain from the sixth week until the end of this work. Compared with the HFD group, the MP-treated mice showed reduced body weight, and accumulation of epididymal, mesentery, and subcutaneous fat tissues (Figures 1A–F). Furthermore, MP intervention could significantly ameliorate the steatosis and damage to the liver. as indicated by decreased levels of ALT in plasma (Figure 1H) and H&E staining of liver tissue (Figures 1K,L). It was observed that HFD could induce extensive liver injury, increased fatty vesicles, and inflammatory cell infiltration, which was significantly reversed by MP intervention. However, MPs showed a limited effect on the accumulation of perirenal fat (Figure 1E) and liver weight (Figure 1G). As shown in Supplementary Figure 1, the food intake and energy intake of the HFD-MP group showed no significant difference from that of the HFD group. Thus, the attenuation of obesity by MP treatment was not due to the reduction in food consumption.

### Mannoproteins treatment improved hyperlipidemia, decreased plasma glucose, and ameliorated systemic inflammation in high-fat diet-induced obese mice

**FIGURE 2:**
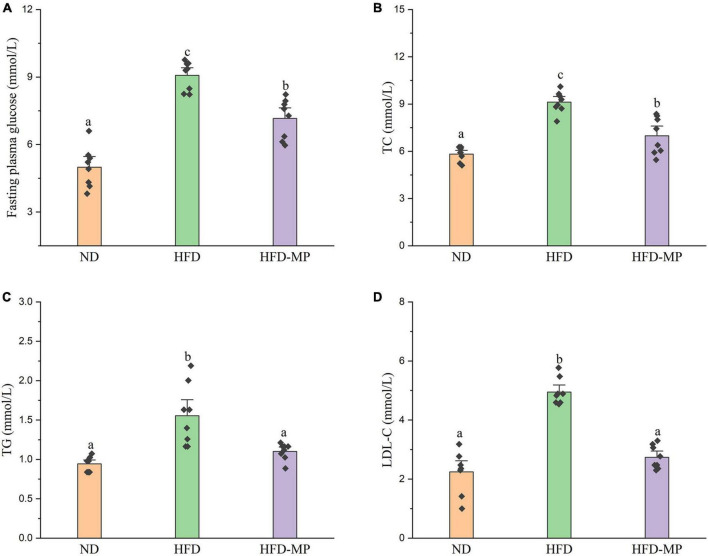
MP intervention reduced HFD-induced high levels of **(A)** fasting plasma glucose, **(B)** TC, **(C)** TG, and **(D)** LDL-C in plasma. The data are represented as the mean ± SEM. Statistical differences were carried out by one-way ANOVA followed by Turkey’s test, *p* < 0.05 indicates significant differences.

**FIGURE 3:**
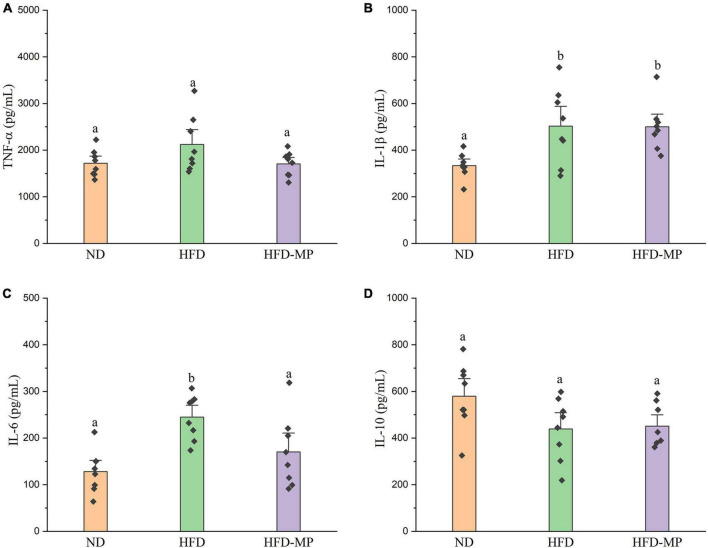
MP intervention attenuated HFD-induced chronic inflammation, including **(A)** TNF-α, **(B)** IL-1β, **(C)** IL-6, and **(D)** IL-10. The data are represented as the mean ± SEM. Statistical differences were carried out by one-way ANOVA followed by Tukey’s test, *p* < 0.05 indicates significant differences.

High-fat diet could significantly increase the level of glucose in plasma, which was reduced after administration of MPs (Figure 2A). HFD usually leads to hyperlipidemia, and thereby increases the potential risk for metabolism-related diseases. Thus, the plasma levels of TC, TG, and LDL-C were also evaluated, and the results showed that high concentrations of TC, TG, and LDL-C induced by HFD were largely reduced by treatment with MPs (Figures 2B–D). Thereinto, the levels of TG and LDL-C in the HFD-MP group showed no significant difference from those in the ND group (*p* > 0.05), suggesting the superior action of MPs for the prevention of hyperlipidemia. Obesity is closely related to chronic low-grade inflammation (30). The pro-inflammatory cytokines in plasma, including TNF-α, IL-1β, IL-6, and IL-10, were detected to evaluate the anti-inflammation effect of MPs in HFD-induced obese mice (Figure 3). It was found that HFD could significantly increase the plasma levels of IL-1β and IL-6, but showed a limited effect on the content of TNF-α and IL-10. MP treatment could reverse the level of IL-6, which showed no significant difference from that in the ND group. However, MP intervention could not change the levels of TNF-α, IL-1β, and IL-10. Furthermore, the effect of MPs on the relative mRNA expression levels of TNF-α, IL-1β, and IL-6 in the liver was investigated (Supplementary Figure 2). It was found that HFD could increase the relative mRNA expression levels of TNF-α, IL-1β, and IL-6, whereas MP treatment could significantly downregulate the mRNA expression levels of TNF-α, IL-1β, and IL-6 (*p* < 0.05).

### Mannoproteins attenuated high-fat diet-induced dysbiosis of gut microbiota

An increasing number of studies have demonstrated that gut microbiota is related to the etiology of obesity and obesity-related complications (31). Thus, it was hypothesized that the gut microbiota was a potential target responsible for the prevention of obesity by MPs. In the present work, the high-throughput sequencing technology was used to systematically analyze the changes in gut microbiota after supplementation with MPs. The Chao1, Richness, Simpson, and Shannon indexes were calculated to quantify the alpha-diversity of gut microbiota, as shown in Supplementary Figure 3. HFD could significantly reduce the Chao1 and Richness indexes. Furthermore, Simpson and Shannon indexes in the HFD group also showed increased or decreased trends compared with those in the ND group with no significant difference. As expected, MPs could reverse these changes of alpha-diversity induced by HFD, by increasing the Chao1, Richness, and Shannon indexes, and decreasing the Simpson index.

**FIGURE 4:**
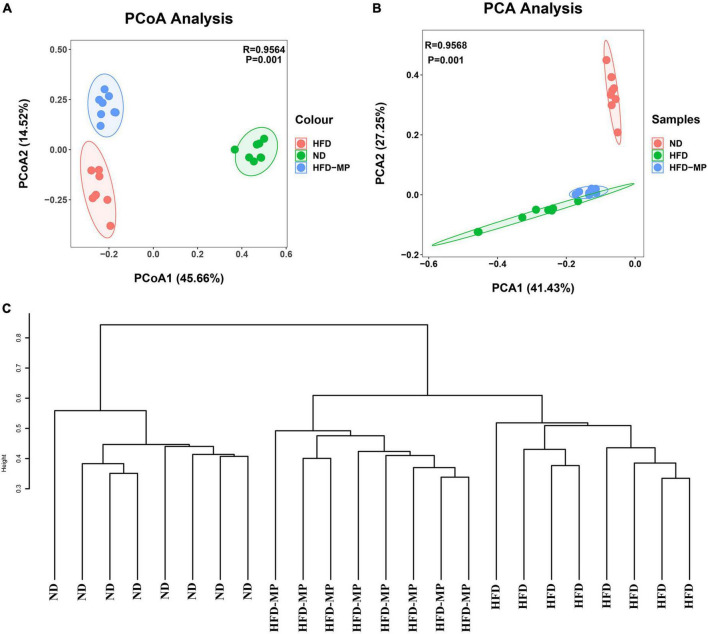
MPs attenuated HFD-induced dysbiosis of gut microbiota evaluated by beta-diversity analyses, including **(A)** PCoA, **(B)** PCA, and **(C)** hierarchical cluster analysis.

**FIGURE 5:**
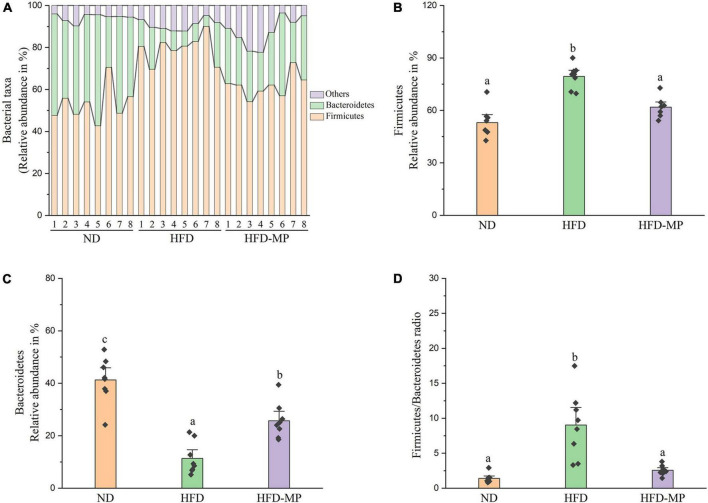
MPs modulate the HFD-disrupted gut microbiota composition at the phylum level. **(A)** Bacterial taxonomic profiling and relative abundances of **(B)** Firmicutes, **(C)** Bacteroidetes, and **(D)** the ratio of Firmicutes to Bacteroidetes. The data are represented as the mean ± SEM. Statistical differences were carried out by one-way ANOVA followed by Tukey’s test, *p* < 0.05 indicates significant differences.

Diet plays an important role in shaping the structure and composition of gut microbiota (32). Principal coordinate analysis (PCoA) was first carried out to visualize the differences in the structure of gut microbiota after HFD and MP treatments. It was found that ND and HFD groups could be clearly distinguished on the basis of the results of PCoA (Figure 4A). Compared with the HFD group, significant separation was also observed after MP treatment, suggesting that MP intervention could change the HFD-treated structure of gut microbiota. Furthermore, principal component analysis (PCA) and hierarchical cluster analysis largely agreed with the result of PCoA (Figures 4B,C). Interestingly, based on the PC1 (41.43%) value in the result of PCA, MPs could lead to a significant shift in the gut microbiota from the HFD group toward the ND group. Thus, MPs significantly modulated the HFD-induced dysbiosis of gut microbiota back to health status. At the phylum level, the gut microbiota of ND, HFD, and HFD-MP groups was all mainly composed of Firmicutes and Bacteroidetes (Figure 5A), which was consistent with the previous works (33, 34). Differently, HFD could significantly increase the relative abundance of Firmicutes and decrease the level of Bacteroidetes compared with the ND group, thereby significantly enhancing the ratio of Firmicutes to Bacteroidetes (Figures 5B–D). MPs could reverse this change induced by HFD treatment, by increasing the relative abundance of Bacteroidetes and decreasing the level of Firmicutes. Furthermore, the ratio of Firmicutes to Bacteroidetes in the HFD-MP group showed no significant difference compared to that observed in the ND group.

**FIGURE 6:**
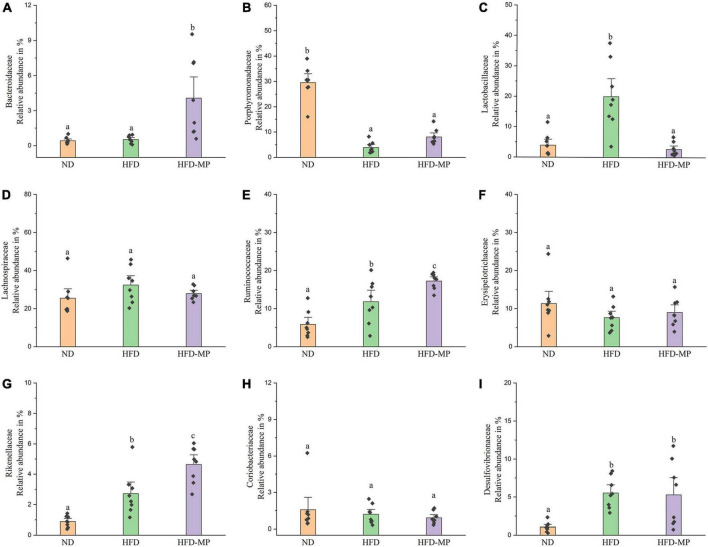
The comparative analysis of nine main families of the gut microbiota, including **(A)** Bacteroidaceae, **(B)** Porphyromonadaceae, **(C)** Lactobacillaceae, **(D)** Lachnospiraceae, **(E)** Ruminococcaceae, **(F)** Erysipelotrichaceae, **(G)** Rikenellaceae, **(H)** Coriobacteriaceae, and **(I)** Desulfovibrionaceae. The data are represented as the mean ± SEM. Statistical differences were carried out by one-way ANOVA followed by Tukey’s test, *p* < 0.05 indicates significant differences.

The gut microbiota at the family level was comparatively analyzed in this work (Figure 6). The result showed that HFD treatment could decrease the relative abundance of Porphyromonadaceae and increase the levels of Lactobacillaceae, Ruminococcaceae, Rikenellaceae, and Desulfovibrionaceae. Compared with the HFD group, MP intervention could significantly increase the relative abundance of Bacteroidaceae, Ruminococcaceae, and Rikenellaceae, and decrease the level of Lactobacillaceae. The gut microbiota at the genus level was also analyzed, as shown in Table 1. Most strikingly, the relative abundance of *Lactobacillus* was increased from 3.94 ± 1.30% to 19.99 ± 3.94% after HFD treatment, which was then decreased to 2.68 ± 0.77% by MP intervention. Likewise, HFD resulted in a significant decrease in the level of *Parabacteroides*, which was reversed by MP treatment. Thus, the modulated effect of MPs on the relative abundance of *Lactobacillus* and *Parabacteroides* may play an important role in the prevention of HFD-induced obesity. Furthermore, MPs could also increase the relative abundance of *Alistipes*, *Bacteroides*, and *Mucispirillum* compared with that in the ND and HFD groups. Then, the relationship between the changed gut microbiota at the genus level by MP and phenotypical changes in obesity was analyzed by Spearman correlation, as shown in Supplementary Figure 4. It was found that *Parabacteroides* and *Alistipes* showed a significant correlation with obesity.

**TABLE 1:** Comparative analysis of the gut microbiota between the groups at the genus level.

	ND	HFD	HFD-MP
*Lactobacillus*	3.94 ± 1.30a	19.99 ± 3.94b	2.68 ± 0.77a
*Barnesiella*	12.65 ± 1.37b	0.00 ± 0.00a	0.00 ± 0.00a
*Desulfovibrio*	0.99 ± 0.22a	5.51 ± 0.72b	5.21 ± 1.51b
*Alistipes*	0.89 ± 0.13a	2.62 ± 0.52b	4.59 ± 0.43c
*Turicibacter*	7.72 ± 1.12b	0.00 ± 0.00a	0.00 ± 0.00a
*Clostridium_XlVa*	2.97 ± 0.79a	1.85 ± 0.43a	2.57 ± 0.24a
*Lactococcus*	0.00 ± 0.00a	4.09 ± 1.55b	1.22 ± 0.22ab
*Bacteroides*	0.42 ± 0.10a	0.52 ± 0.11a	4.07 ± 1.21b
*Parabacteroides*	2.28 ± 0.43b	0.39 ± 0.12a	2.10 ± 0.20b
*Helicobacter*	0.66 ± 0.28a	0.62 ± 0.19a	2.25 ± 0.75a
*Mucispirillum*	0.40 ± 0.15a	0.82 ± 0.37ab	2.14 ± 0.64b
*Oscillibacter*	0.80 ± 0.23a	0.94 ± 0.21ab	1.56 ± 0.13b
*Roseburia*	0.00 ± 0.00a	1.49 ± 0.51b	1.07 ± 0.39ab
*Romboutsia*	0.02 ± 0.01a	1.29 ± 0.3b	0.38 ± 0.21a
*Clostridium_XlVb*	0.25 ± 0.05a	0.57 ± 0.09ab	0.81 ± 0.13b
*Enterorhabdus*	0.78 ± 0.36a	0.19 ± 0.06a	0.16 ± 0.04a
*Bifidobacterium*	0.87 ± 0.22b	0.25 ± 0.07a	0.00 ± 0.00a
*Prevotella*	1.10 ± 0.26b	0.00 ± 0.00a	0.00 ± 0.00a
*Acetatifactor*	0.07 ± 0.03a	0.29 ± 0.11ab	0.35 ± 0.06b
*Clostridium_sensu_stricto*	0.60 ± 0.15b	0.00 ± 0.00a	0.06 ± 0.02a
*Clostridium_IV*	0.32 ± 0.05b	0.16 ± 0.05a	0.14 ± 0.03a
*Clostridium_XVIII*	0.03 ± 0.01a	0.2 ± 0.06ab	0.34 ± 0.09b
*Odoribacter*	0.10 ± 0.05a	0.38 ± 0.19a	0.07 ± 0.05a
*Olsenella*	0.17 ± 0.09ab	0.03 ± 0.01a	0.27 ± 0.07b
*Akkermansia*	0.40 ± 0.24a	0.00 ± 0.00a	0.00 ± 0.00a
*Ruminococcus*	0.37 ± 0.08b	0.00 ± 0.00a	0.00 ± 0.00a
*Streptococcus*	0.01 ± 0.00a	0.29 ± 0.10b	0.07 ± 0.02a
*Parasutterella*	0.27 ± 0.06b	0.00 ± 0.00a	0.00 ± 0.00a
*Anaerotruncus*	0.01 ± 0.00a	0.13 ± 0.06a	0.12 ± 0.04a
Others	60.91 ± 1.74	57.41 ± 3.27	67.75 ± 2.29

The data are represented as the mean ± SEM. Statistical differences were carried out by one-way ANOVA followed by Tukey’s test, *p* < 0.05 indicates significant differences.

The different letters indicate significant differences between the groups (*p* < 0.05).

**FIGURE 7:**
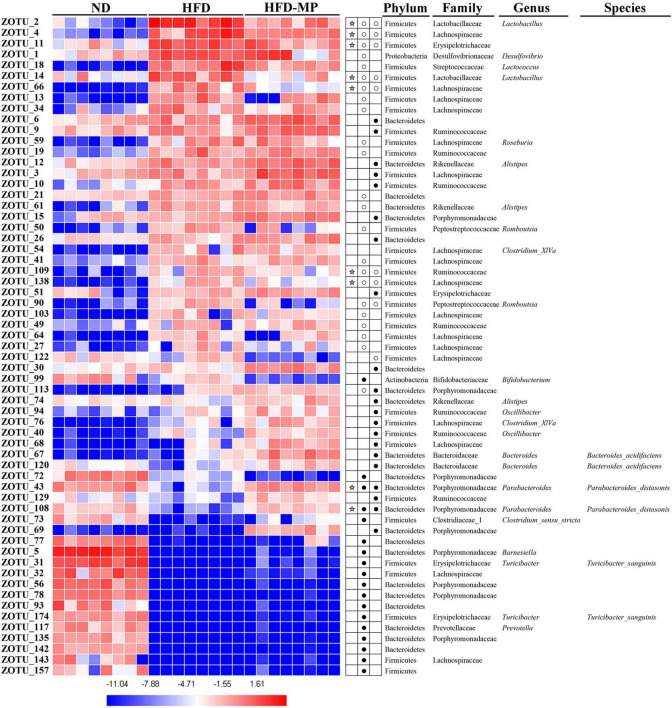
Gut microbiota composition was altered by HFD or MP intervention at the ZOTUs levels. Heatmap shows the relative abundance of 62 ZOTUs (ln transformed). The dots (●) and circles (○) showed the more or less relative abundances of ZOTUs in ND or MPs groups compared with the HFD group. The star (☆) represented ZOTUs changed by HFD but were reversed after treatment with MPs. Statistical differences were carried out by one-way ANOVA followed by Tukey’s test, *p* < 0.05 indicates significant differences.

**FIGURE 8:**
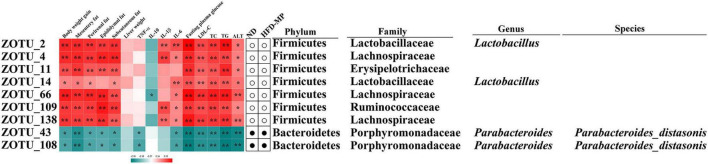
Nine ZOTUs reversed by MP intervention were significantly correlated with features of obesity. The heatmap shows the *R*-value of Spearman’s correlation between the relative abundance of ZOTUs and features of obesity. The dots (correlation between the more or less relative abundance of ZOTUs in the ND or MP groups compared with the HFD group. * and **Show the significant associations (*p* < 0.05 and *p* < 0.01, respectively) based on Spearman’s correlation analysis.

The different gut microbiota at the same genus level may show different responses after treatment with MPs, thus the gut microbiota at the ZOTU level was analyzed to further excavate the key gut microbiota contributing to the prevention of obesity. The ZOTUs with a relative abundance of more than 0.1% were used to proceed with further analysis. As shown in Figure 7, HFD and MPs could significantly change 62 ZOTUs compared with the ND group. HFD could lead to 41 changed ZOTUs, including increasing 23 ZOTUs and decreasing 18 ZOTUs. Thereinto, nine ZOTUs were found to be significantly reversed by MP intervention. Then, the relationship between the relative abundance of these reversed ZOTUs and phenotypical changes of obesity was analyzed by Spearman correlation (Figure 8), and the result showed that seven ZOTUs were positively corrected with obesity, and two ZOTUs were negatively associated with obesity. Thereinto, two ZOTUs (ZOTU2 and ZOTU14) belonging to *Lactobacillus* and two ZOTUs (ZOTU43 and ZOTU108) belonging to *Parabacteroides distasonis* may play a key role in the prevention of obesity.

### Mannoproteins increased the content of acetic acid in high-fat diet-fed mice

The SCFAs are the main metabolites produced by the fermentation of polysaccharides by gut microbiota, which are speculated to play an important role in the biological activities of polysaccharides (35). Thereinto, acetic acid, propionic acid, and butyric acid are the most abundant SCFAs in the human body and colon, whereas other SCFAs, such as formate, valerate, and caproate, are minor end products in the colon (36). Thus, the levels of acetate, propionate, and butyrate were measured in different groups, and the result showed that HFD significantly reduced the levels of acetate, propionate, and butyrate in the cecal contents of mice (Supplementary Figure 5). The MP intervention could lead to a significant increase in the level of acetic acid from 3.28 ± 0.22 mmol/g to 7.84 ± 0.96 mmol/g; however, MPs showed a limited effect on the content of propionic acid and butyric acid. Furthermore, the effect of MPs on the mRNA expression of G protein-coupled receptors (GPRs), including GPR43 and GPR41, in the liver was investigated to further verify the key role of SCFAs in the attenuation of obesity (Supplementary Figure 6). The result showed that HFD treatment significantly reduced the mRNA expression of GPR41 and GPR43, whereas MP intervention could upregulate the expression of GPR41 and GPR43.

## Discussion

Obesity has become a leading public health problem with pandemic proportions and can further increase the rates of complications, such as cardiovascular disease and type 2 diabetes (T2D) (37). An increasing number of studies have demonstrated that gut microbiota populations are sensitive to genetic, environmental, and diet influences, and hence can directly or indirectly affect the energy balance and energy stores (12). Thus, the gut microbiota is expected as a promising target for the prevention and treatment of obesity (38). Polysaccharides, serving as a superior prebiotic, could modulate the gut microbiota by selectively stimulating the growth of beneficial bacteria and inhibiting the harmful microbiota, thereby improving host health (39). Furthermore, polysaccharides could also modulate the metabolism of probiotics (40). MP is one of the most important components of yeast cell walls. The previous work has shown that MPs could stimulate angiogenesis (27) and had immunoactivities and antineoplastic activities (26). However, the effects of MPs on obesity and gut microbiota are still unknown. Here, the HFD-induced obesity mice model was used to investigate the potential anti-obesogenic effect of MPs. Over 10 weeks of treatment, HFD could significantly induce obesity in the mice model. As expected, MPs significantly prevented HFD-induced body weight gain, fat accumulation, and liver steatosis. Furthermore, the levels of glucose, TC, TG, and LDL-C in plasma were also ameliorated by the treatment with MP. Thus, the results in the present work demonstrated that MP intervention could reduce obesity and metabolic disorders in HFD-fed mice. However, it would be more convincing if the dose setting or positive control was involved in this work.

It has been reported that HFD could damage the gut integrity and lead to a leaky gut, and the endotoxin lipopolysaccharide (LPS) released from Gram-negative bacteria in the gut enter the bloodstream, thereby leading to metabolic inflammation in obese mice (30). Thus, HFD-induced obesity is usually associated with chronic, low-grade inflammation. In the present work, IL-1β, and IL-6 levels were significantly enhanced after HFD treatment, and MPs could significantly reduce the level of IL-6, which showed no significant difference from those in the ND group. The decrease in the pro-inflammatory cytokines by the dietary polysaccharides contributing to the prevention of metabolic diseases has been widely reported. For example, an insoluble polysaccharide from the sclerotium of *Poria cocos* could reduce the plasmatic TNF-α in ob/ob mice (41). Likewise, the polysaccharides isolated from *Hirsutella sinensis* decreased the serum levels of the pro-inflammatory cytokines IL-1β and TNF-α in the HFD-fed mice (42). Thus, the improvement of metabolic disorders by MPs might be related to the suppression of chronic inflammation.

The accumulating evidence has demonstrated that dietary habit, especially a HFD, could lead to dysbiosis of gut microbiota, which might thereby lead to some pathologic conditions of obesity and obesity-related complications. Recently, some foods or food additives, such as processed foods (43), dietary emulsifiers (44), and artificial sweeteners (45), could promote metabolic diseases by disordering the gut microbiota. Alpha-diversity, including Chao1, Richness, Simpson, and Shannon indexes, could reflect the diversity and richness of bacteria (46). In this work, HFD could significantly affect the structure and composition of gut microbiota, which is evidenced by decreasing alpha-diversity and changing beta-diversity indexes. The reports in animal and clinical studies showed that decreased alpha-diversity and richness values were observed in obese subjects and animals (28, 47, 48). MP treatment could not only increase the alpha-diversity, including Chao1, Richness, Simpson, and Shannon indexes, but could also change the structure of gut microbiota from the HFD group toward the ND group based on beta-diversity.

The result at the phylum level indicated that the gut microbiota was dominated by Firmicutes and Bacteroidetes. The ratio of Firmicutes to Bacteroidetes has been reported to relate to metabolic diseases, and high levels of Firmicutes and low levels of Bacteroidetes were observed in obese humans and animals (49–51). Thus, the decrease in the ratio of Firmicutes to Bacteroidetes may contribute to the prevention of obesity and metabolic diseases. A decrease in the ratio of Firmicutes to Bacteroidetes by anti-obesogenic candidates was widely reported (52, 53). In this work, a similar trend toward a decreased ratio of Firmicutes to Bacteroidetes was obtained after MP treatment, which may contribute to the prevention of obesity by MPs. At the family level, the MPs induced increased levels of Bacteroidaceae, Ruminococcaceae, and Rikenellaceae. Therefore, the relative abundance of Bacteroidaceae has been reported to negatively link to obesity (54, 55). Furthermore, the level of Bacteroidaceae is determined by SCFA-producing bacteria (56), which could be regarded as a positive outcome predictor of individual weight loss (57). Lactobacillaceae was usually considered as beneficial bacteria in the gut for the prevention of obesity (58, 59). On the other hand, some reports showed that Lactobacillaceae has a positive relationship with obesity (60, 61). The different bacteria in the same family may play different roles in the metabolic phenotype of obesity, thus the gut microbiota was also analyzed at the genus or ZOTU levels.

It was found that *Lactobacillus*, belonging to Lactobacillaceae, and *Parabacteroides*, belonging to Porphyromonadaceae, were significantly reversed by MP treatment. Furthermore, *Alistipes* (belonging to Rikenellaceae) which was increased by MPs showed a significant correlation with obesity. To further identify species-level phylotypes or specific bacterial taxa contributing to the prevention of obesity by MPs, the gut microbiota was analyzed at the ZOTU level. *Lactobacillus* (ZOTU2 and ZOTU14), which was positively corrected with obesity, and *P. distasonis* (ZOTU43 and ZOTU108), which showed negative relation to obesity, were significantly reversed after MP intervention. *P. distasonis*, regarded as one of the 18 core members in the human gut microbiota, plays an important role in human health (62). The lower level of *P. distasonis* has been observed in patients and animals with metabolic diseases (63, 64). Furthermore, the alleviation of obesity and obesity-related dysfunctions by *P. distasonis* has been reported, which was due to the generation of succinate and secondary bile acids in the gut (14). Moreover, the other health-promoting functions of *P. distasonis*, such as blocking colon tumor formation (65) and alleviating colitis (66), have also been widely reported. Thus, *P. distasonis* has been regarded as potential probiotic for improving our health (67). The previous work showed that polysaccharides, such as inulin, could promote the proliferation of *P. distasonis* and thereby improve human health (68). In this work, the MPs could also increase the level of *P. distasonis* in HFD-induced obese mice contributing to the prevention of obesity. *Lactobacillus* showed a particularly interesting role in this work. It is well known that lots of species in the genus *Lactobacillus* are probiotic bacteria (69, 70), which can reduce the risk of metabolic diseases. On the other hand, a report showed that some species belonging to *Lactobacillus*, such as *Lactobacillus reuteri*, were positively associated with obesity, while others were related to normal weight (71). Recently, a systematic review of randomized controlled clinical trials summarized the effect of *Lactobacillus* on obesity, and it was found that the beneficial or detrimental effects of *Lactobacillus* on obesity are strain-dependent (72). Thus, the prevention of obesity by MPs might be related to the inhibition of *Lactobacillus*. Unfortunately, the species for *Lactobacillus* in this work could not be identified by sequencing, which should be further investigated.

SCFAs, the key metabolites produced by gut microbiota, play an important role in improving colonic and systemic health (35), which could help to explain why and how the changes in gut microbiota contribute to human health and diseases (73). A growing amount of evidence suggests that SCFAs could enter into the bloodstream, and thereby affect the tissues and organs beyond the gut (74). Therefore, the specific species, diversity, and absolute amount of gut microbiota play a key role in the production of SCFAs (75). The diet intervention could alter either the bacterial species or the bacterial biosynthetic enzymes, thereby leading to alterations in microbial SCFA production (76). A potential strategy based on the modulation of gut microbiota by prebiotics has been presented to stimulate the production of SCFAs, thereby preventing diseases and improving human health (77, 78). Thus, we suspected that the SCFAs would be changed due to the modulation of gut microbiota by MP, which thereby contributes to the prevention of obesity. In this work, MPs could increase the level of acetate decreased by HFD treatment, whereas they showed limited effects on the contents of propionate and butyrate. Furthermore, the mRNA expression of GPR41 and GPR43 in the liver was significantly upregulated by MPs, suggesting that SCFAs played a key role in the prevention of obesity by MPs. A lot of reports have shown that acetate administration could reduce body weight, decrease hepatic fat accumulation, and improve insulin sensitivity in HFD-fed mice (79, 80). Likewise, SCFA intervention studies in humans also further demonstrated that consumption of acetate could significantly reduce the body weight of patients with obesity (81). SCFAs could activate GPR41 and GPR43 to improve immune responses, and the activation of GPR41/43 could further modulate the levels of pro-inflammatory factors. It has been reported that polysaccharides-derived SCFAs could significantly reduce the level of pro-inflammatory factors, such as LPS in the blood. Furthermore,SCFA might also directly reverse LPS-induced inflammation (75, 82). In addition, acetate can significantly regulate the levels of DNA methylation at the host miR-378a promoter, which also contribute to the improvement of obesity and glucose intolerance (83). It has been reported that Parabacteroides could utilize polysaccharides with its glycoside hydrolase, and further produced acetate to affect host health (84, 85). It is expected to be a potential strategy to increase the level of acetate by prebiotics, thereby preventing and treating obesity. Thus, the increased level of acetate by MPs may also contribute to the prevention of obesity in this work. In addition to SCFAs, the other metabolites were not measured in the present study, which could be further investigated by metabolomics in our next work (86).

## Conclusion

In conclusion, the HFD-induced obese mice model was used to investigate the potential anti-obesogenic effect of MPs and its potential mechanism. The result showed that MPs significantly attenuated HFD-induced obesity. MPs could not only increase the alpha-diversity of gut microbiota, but also change the structure of gut microbiota from the HFD group to the ND group. Furthermore, harmful *Lactobacillus* and probiotic *P. distasonis* may be potential key gut microbiota responsible for the prevention of obesity by MPs. This preliminary research showed promise for the efficacy of MPs in the prevention of HFD-induced obesity, thus MPs were expected to serve as a functional food for the improvement of human health.

## Data availability statement

The original contributions presented in this study are publicly available. This data can be found here: Genome Sequence Archive in the BIG Data Center Chinese Academy of Sciences (https://ngdc.cncb.ac.cn/gsa/) with BioProject number of PRJCA009982 under accession codes of CRA007210.

## Ethics statement

All animal experiment protocols were approved by the Institutional Animal Ethics Committee of Experimental Animal Center of Nanjing Agricultural University.

## Author contributions

XL, JSW, JLW, GC, and XZ contributed to the conception and design of the study. YK organized the database. DC performed the statistical analysis. XL wrote the first draft of the manuscript. GC and XZ wrote sections of the manuscript. All authors contributed to manuscript revision, read, and approved the submitted version.

## Footnotes

1
http://drive5.com
