# Supplementary material for: Yeast mannoproteins are expected to be a novel potential functional food for attenuation of obesity and modulation of gut microbiota
Source: Front Nutr. 2022 Oct 14;9:1019344. doi: 10.3389/fnut.2022.1019344 (PMC9614242; doi:10.3389/fnut.2022.1019344)
Supplement: Supplementary file 1 [file Data_Sheet_1.pdf]

**Yeast mannoproteins is expected as a novel potential functional food for attenuation of obesity and modulation of gut microbiota**

Xiang Li,[a] Junsong Wu,[b] Yijun Kang,[a] Dan Chen,[c] Guijie Chen,[c]* , Xiaoxiong Zeng,[c]* Jialian Wang[a]

[a]School of Marine and Biological Engineering, Yancheng Teachers' University, Yancheng, China

[b]Department of Basic Medical Science, Jiangsu Vocational College of Medicine, Yancheng 224005, Jiangsu, China,

[c]College of Food Science and Technology, Nanjing Agricultural University, Nanjing 210095, Jiangsu, China

* Corresponding authors. E-mail addresses: guijiechen@njau.edu.cn (G. Chen), zengxx@njau.edu.cn (X. Zeng).

**Table S1. Primer sequences for RT-qPCR experiments**

| Gene | Forward primer (5'-3') | Reverse primer (5'-3') |
|---|---|---|
| TNF-α | CTCATGCACCACCATCAAGG | ACCTGACCACTCTCCCTTTG |
| IL-1β | AGCTTCAAATCTCGCAGCAG | TCTCCACAGCCACAATGAGT |
| IL-6 | GACTGATGCTGGTGACAACC | AGACAGGTCTGTTGGGAGTG |
| GPR41 | CGACTAGAGATGGCTGTGGT | AGAAGATGAGCAGTGTGGCT |
| GPR43 | TCCAGCCTGGCTTTCCAATA | GCCTGCAGGAGACATTTCAG |
| GAPDH | GGACTTACAGAGGTCCGCTT | CTATAGGGCCTGGGTCAGTG |

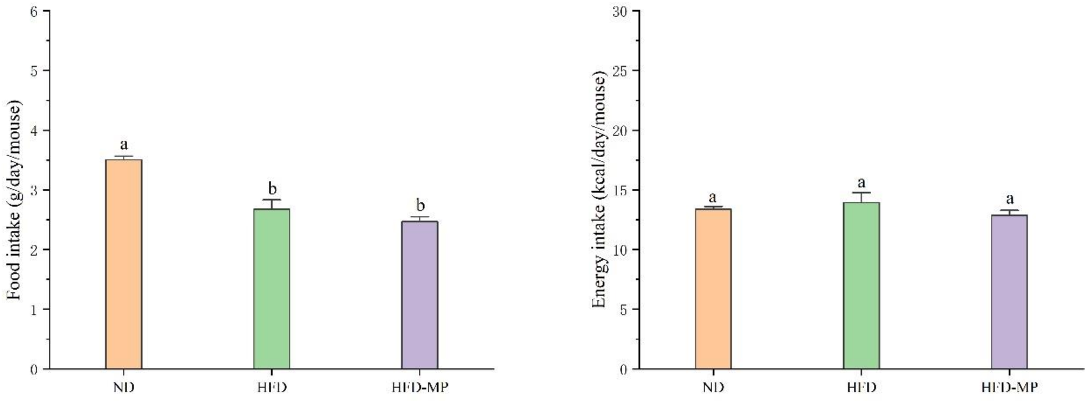


**Fig. S1.** Average food intake (a) and energy intake (b) per day of mice. The data were represented as the mean ± SEM. Statistical differences were carried out by one-way ANOVA followed by Tuckey test, $p < 0.05$ indicates significant.

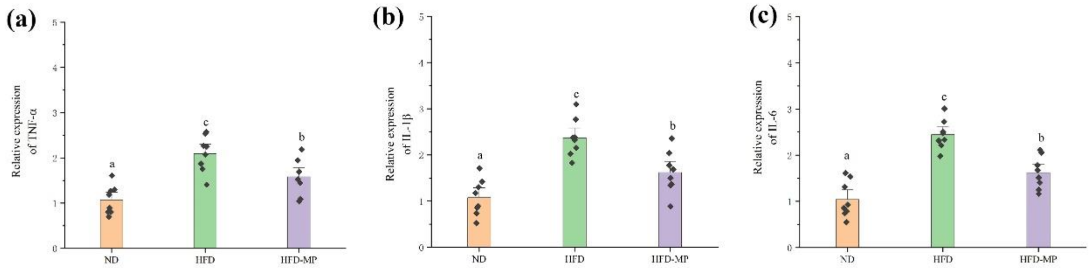


**Fig. S2.** The effect of MPs on the relative mRNA expression levels of TNF-α (a), IL-1β (b) and IL-6 (c) in liver. The data were represented as the mean ± SEM. Statistical differences were carried out by one-way ANOVA followed by Tuckey test, $p < 0.05$ indicates significant.

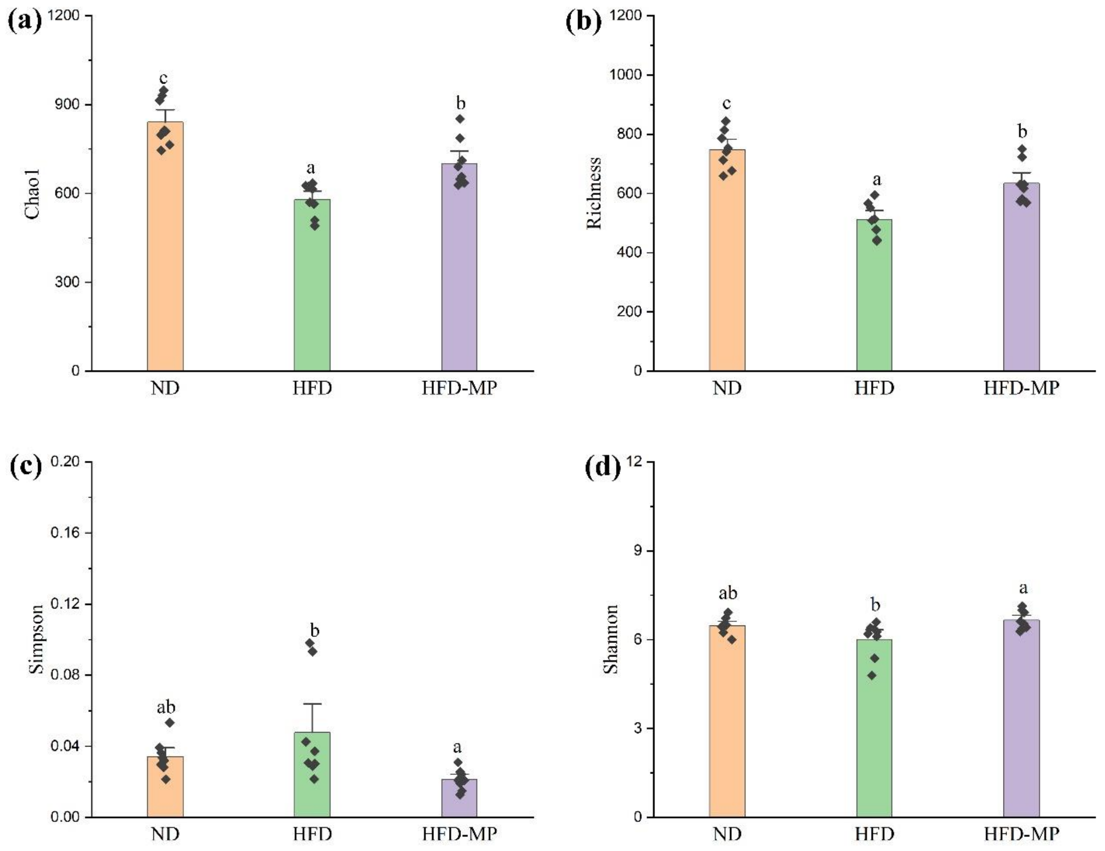


**Fig. S3.** Effect of MPs on the alpha diversity of gut microbiota, including (a) Chao1, (b) Richness, (c) Simpson and (d)Shannon indexes. The data were represented as the mean ± SEM. Statistical differences were carried out by one-way ANOVA followed by Tuckey test, $p < 0.05$ indicates significant.

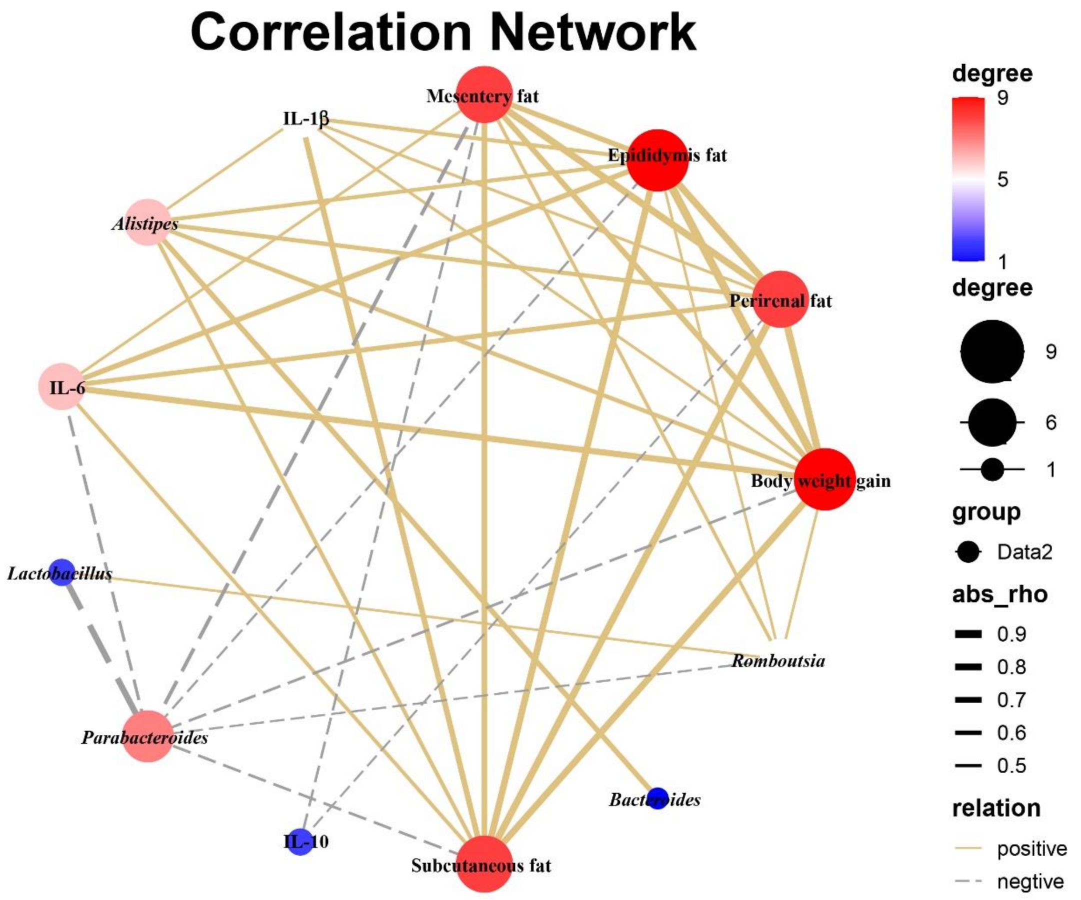


**Fig. S4.** Spearman's correlation between relative abundances of key gut microbiota at the genus level and features of obesity.

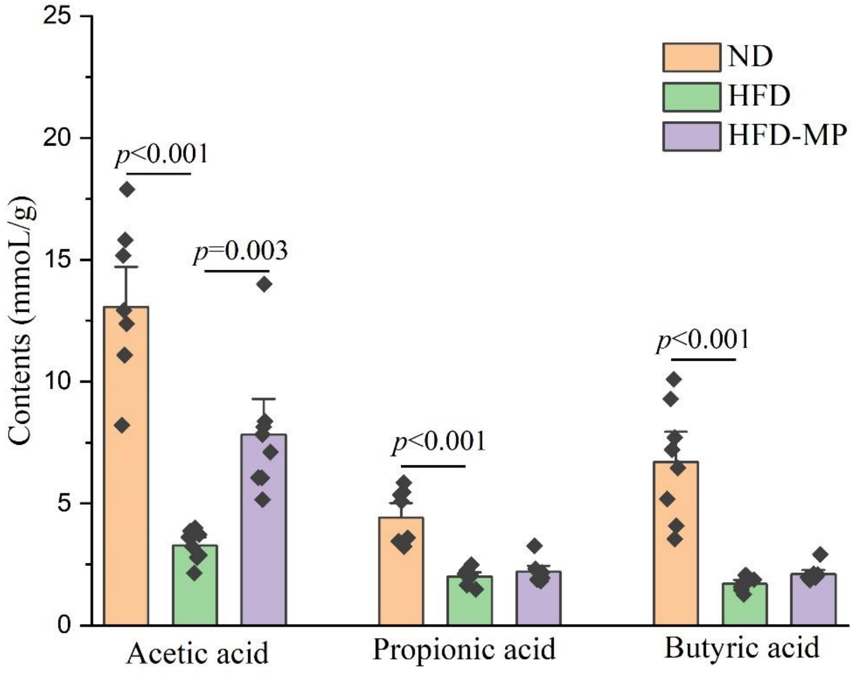


**Fig. S5.** The effect of MPs on the levels of SCFAs in mice cecal contents. The data were represented as the mean ± SEM. Statistical differences were carried out by one-way ANOVA followed by Tuckey test, $p < 0.05$ indicates significant.

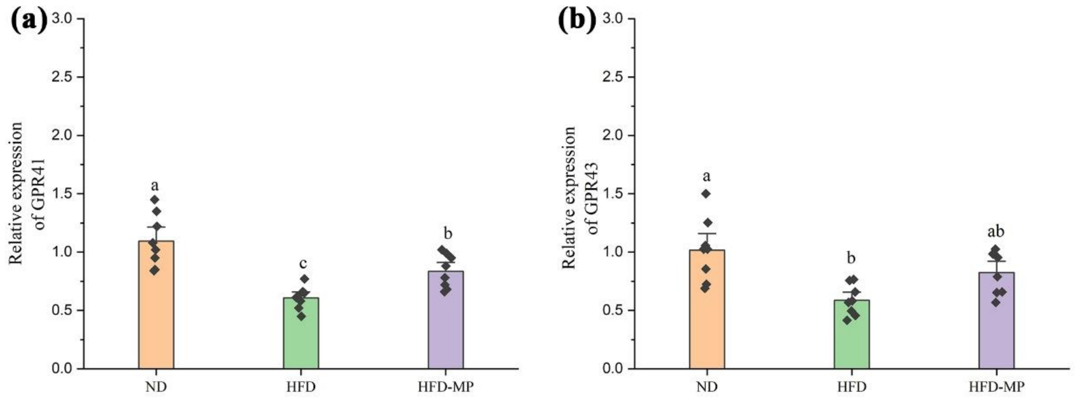


**Fig. S6.** The effect of MPs on the relative mRNA expression levels of GPR41 (a) and GPR43 (b). The data were represented as the mean ± SEM. Statistical differences were carried out by one-way ANOVA followed by Tuckey test, $p < 0.05$ indicates significant.
